# shRNAs targeting mouse *Adam10* diminish cell response to proinflammatory stimuli independently of *Adam10* silencing

**DOI:** 10.1242/bio.059092

**Published:** 2022-03-04

**Authors:** Maria Czarnek, Krystyna Stalińska, Katarzyna Sarad, Joanna Bereta

**Affiliations:** Department of Cell Biochemistry, Faculty of Biochemistry, Biophysics and Biotechnology, Jagiellonian University in Kraków, Gronostajowa 7, 30-387 Kraków, Poland

**Keywords:** RNA interference, shRNA, Off-target effects, ADAM10, ADAM17

## Abstract

RNA interference is one of the common methods of studying protein functions. In recent years critical reports have emerged indicating that off-target effects may have a much greater impact on RNAi-based analysis than previously assumed. We studied the influence of *Adam10* and *Adam17* silencing on MC38CEA cell response to proinflammatory stimuli. Eight lentiviral vector-encoded shRNAs that reduced ADAM10 expression, including two that are specific towards ADAM17, caused inhibition of cytokine-induced *Nos2* expression presumably via off-target effects. ADAM10 silencing was not responsible for this effect because: (i) CRISPR/Cas9 knockdown of ADAM10 did not affect *Nos2* levels; (ii) ADAM10 inhibitor increased rather than decreased *Nos2* expression; (iii) overexpression of ADAM10 in the cells with shRNA-silenced *Adam10* did not reverse the effect induced by shRNA; (iv) shRNA targeting ADAM10 resulted in decrease of *Nos2* expression even in ADAM10-deficient cells. The studied shRNAs influenced transcription of *Nos2* rather than stability of *Nos2* mRNA. They also affected stimulation of *Ccl2* and *Ccl7* expression. Additionally, we used vectors with doxycycline-inducible expression of chosen shRNAs and observed reduced activation of NF-κB and, to a lesser extent, AP-1 transcription factors. We discuss the requirements of strict controls and verification of results with complementary methods for reliable conclusions of shRNA-based experiments.

## Abstract (precis)

**Summary:** Use of several specific shRNAs is not enough to escape a pitfall of their off-target activity: the case of *Adam10* and *Adam17* silencing.

## INTRODUCTION

ADAM10 and ADAM17 are two prominent and most closely related members of ADAM (a disintegrin and metalloprotease) family, indispensable for normal growth, differentiation and homeostasis of the organism including the immune system function (Lambrecht et al., 2018; Scheller et al., 2011; Weber and Saftig, 2012). They play a role of sheddases and release ectodomains of more than a hundred membrane proteins, such as growth factors, cytokines, receptors, adhesion molecules and others. Thus, they provide active mediators, modulate cell–cell and cell–tissue interactions, influence cell responsiveness to environmental stimuli, and generate substrates for intramembrane proteases, which then produce transcriptional co-activators (Edwards et al., 2008; Groot and Vooijs, 2012; Maretzky et al., 2005; Zunke and Rose-John, 2017).

Although ADAM10 and ADAM17 show overlapping substrate specificity, there are membrane proteins preferentially shed by one of them. For example, ADAM10 is a major sheddase for Notch (Groot and Vooijs, 2012), ephrin (Mancia and Shapiro, 2005), FasL (Schulte et al., 2007), EGF and betacellulin (Sahin et al., 2004), and mouse NRG2 (Czarnek and Bereta, 2020), and ADAM17 for TNF (Black et al., 1997; Moss et al., 1997), both TNF receptors (Peschon et al., 1998), L-selectin (Condon et al., 2001), IL6R (Schumacher and Rose-John, 2019), TGFα, amphiregulin, and HB-EGF (Sahin et al., 2004). The activities of ADAM10 and ADAM17 are regulated by complex mechanisms, including influence of membrane lipids (Reiss and Bhakdi, 2017; Sommer et al., 2016) and interactions with distinct protein partners, tetraspanins C8 and iRhoms, respectively (Matthews et al., 2017). Both ADAM10 and 17 are involved in progression of various tumors and are considered potential targets for cancer therapies (Saha et al., 2019; Smith et al., 2020; Zunke and Rose-John, 2017). The distinction between common and selective ADAM10 and ADAM17 substrates and regulatory mechanisms specific for each protease is of scientific and therapeutic importance.

*In vitro* studies of ADAM10 and ADAM17 functions often use cell lines derived from conditional knockout or hypomorphic mice (Chalaris et al., 2010; Horiuchi et al., 2007; Jorissen et al., 2010; Zbodakova et al., 2021). Alternatively, cell lines with silenced expression of ADAM10 or ADAM17 by RNA interference are utilized. This approach circumvents the limitations of the availability of ADAM knockouts in certain cell lineages and tumor cell lines and is facilitated by the availability of commercial vectors encoding specific shRNAs.

We have previously generated MC38CEA and P388D1 cell lines, in which ADAM17 expression had been silenced by stable transfection of the cells with a plasmid coding for shRNA targeting ADAM17 (Das et al., 2012). In those cells the levels of cytokine- or LPS-induced expression of inducible nitric oxide synthase, a recognized marker of inflammation, were substantially lower than in their ADAM17-proficient counterparts (Fig. S1). This effect could not be easily explained by reduced shedding of known ADAM17 substrates, as proinflammatory stimuli were provided externally and diminished shedding of receptors should result in the increase rather than decrease of cell activation. Therefore, we decided to investigate the mechanism behind this phenomenon in cell lines, in which ADAM17 would be silenced by shRNA encoded by viral vectors.

Short hairpin RNA (shRNA) expression vectors, including retroviral and lentiviral vectors, utilize the natural mechanism of gene silencing by miRNA derived from endogenous precursors (Paddison et al., 2004; Shi, 2003). An expression cassette encoding specific shRNA is stably integrated into the DNA of transduced cells. After transcription shRNA is exported from the nucleus and processed by Dicer to an siRNA duplex. Argonaute protein (AGO) is then loaded with one strand of the duplex and together with other proteins forms an RNA-induced silencing complex (RISC), which binds to a target mRNA resulting in inhibition of mRNA translation and/or its degradation (Paddison et al., 2004; Sheng et al., 2020). In recent years, shRNA-mediated gene silencing attracted growing criticism, mainly due to off-target effects that frequently led to false conclusions (Housden and Perrimon, 2016; Lin et al., 2019; Lin et al., 2017). To minimize this threat, it is recommended to use several different silencing sequences. Commercial libraries, such as MISSION^®^, usually offer vectors encoding five different, specific shRNAs for each gene. Their specificity is verified by bioinformatic data (Merck, MISSION™ shRNA Library: Next Generation RNA Interference. 2021, https://www.sigmaaldrich.com/PL/pl/technical-documents/technical-article/genomics/gene-expression-and-silencing/mission-shrna-library).

Our work pointed to the possible pitfalls of shRNA-mediated silencing of gene expression. We found that certain commercial ADAM17-targeting sequences may silence ADAM10 expression as well. Also, the high number of specific gene-targeting shRNAs does not guarantee that observed effects result from gene silencing. We found diminished responsiveness to proinflammatory cytokines of MC38CEA cells transduced with a number of ADAM10-targeting shRNAs, but this phenomenon did not result from ADAM10 deficiency.

## RESULTS

**Fig. 1.**
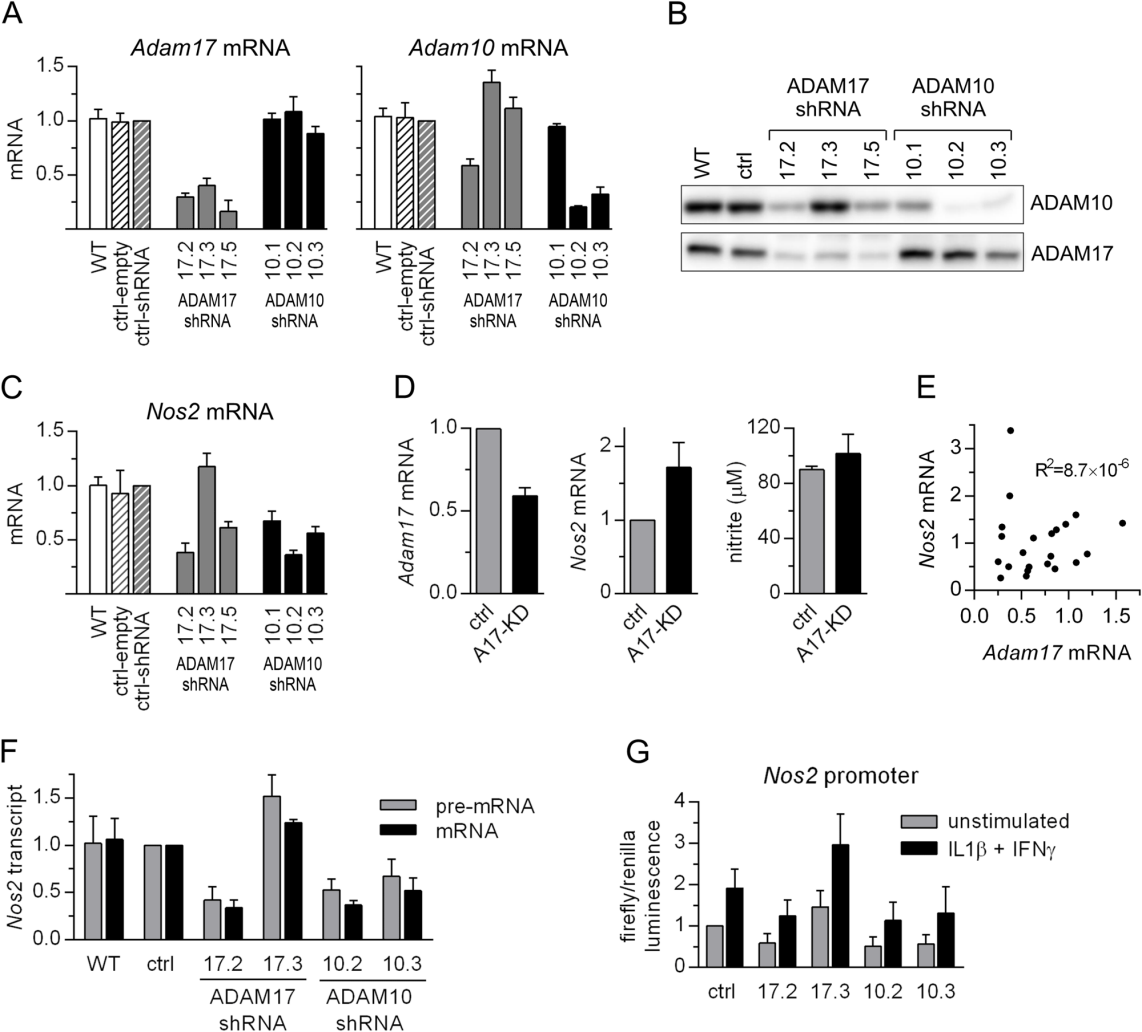
**shRNAs targeting *Adam17* or *Adam10* diminish cytokine-induced *Nos2* expression.** The cells were stimulated with IL1β and IFNγ for 6 h (for RT-qPCR analysis) or for 20 h (for nitrite levels measurement). (A,C) RT-qPCR analysis of *Adam17*, *Adam10*, and *Nos2* mRNA levels in wild-type (WT) MC38CEA cells or MC38CEA cells transduced with one of lentiviral vectors (MISSION^®^): empty one (ctrl-empty) or coding for: non-targeting shRNA (ctrl-shRNA) or one of the *Adam17- or Adam10-*targeting shRNA sequences. The relative levels of the transcripts in control cells were taken as 1. Data are shown as MV±s.d. from three (*Adam17*), four *(Adam10*), and five (*Nos2*) independent experiments. (B) Western blot (WB) analysis of ADAM17 and ADAM10 levels in MC38CEA cells described in A. Representative images of WB of three independent experiments are shown. Whole WB and Ponceau S-stained membranes are given in Fig. S2. (D) RT-qPCR analysis of *Adam17* and *Nos2* (left and middle panels) and levels of nitrite (right panel) in cultures of whole population of MC38CEA cells, in which *Adam17* was targeted by CRISPR/Cas9. In control cells *EGFP*-specific sgRNA was used instead of *Adam17*-specific sgRNAs. The relative levels of transcripts in control cells were taken as 1. MV±s.d. from five (*Adam17*), six (*Nos2*) and three (nitrite) independent experiments are shown. (E) Analysis of correlation between *Adam17* and *Nos2* transcript levels in independent clones derived from *Adam17*-knockdown population. Source data are presented in Fig. S4. (F) RT-qPCR analysis of the levels of precursor and mature *Nos2* mRNA in MC38CEA cells described in A. The relative levels of the transcripts in control cells were taken as 1. Data are shown as MV±s.d. from three independent experiments. (G) Relative luminescence signals in MC38CEA cells described in (A) transfected with a plasmid containing luciferase CDS under *Nos2* promoter. Luminescence signals of unstimulated, control cells were taken as 1. Data are shown as MV±s.d. from four independent experiments.

We have undertaken research to elucidate the mechanism behind diminished responsiveness of cells with silenced expression of ADAM17 to proinflammatory stimuli. We generated MC38CEA and P388D1 cell lines, in which ADAM17 was silenced with lentiviral vectors carrying various shRNAs targeting *Adam17*. The advantage of lentiviral vectors is that, thanks to efficient gene transfer, the resulting cell lines came from the pool of cells instead of individual clones as in the case of plasmid vectors previously used in our research (Fig. 1). Three out of five commercial shRNA sequences (hereinafter referred to as A17.2, A17.3, and A17.5 and presented in Table S1) significantly silenced ADAM17 expression in MC38CEA (Fig. 1A,B). In the case of A17.2 and A17.5, diminished expression of ADAM17 was associated with diminished cytokine-induced expression of *Nos2* (Fig. 1C). Silencing of ADAM17 was less efficient in P388D1 than in MC38CEA cells but was also accompanied by decreased *Nos2* inducibility (Fig. S3).

However, when ADAM17 expression was knocked down in MC38CEA cells by CRISPR/Cas9 gene editing, the diminished level of *Adam17* observed in an entire cell population was not accompanied by decreased expression and activity of iNOS (Fig. 1D). In individual clones derived from the CRISPR/Cas9-edited MC38CEA population the levels of *Adam17* mRNA were decreased by 40–70% compared with the mean value of *Adam17* mRNA levels in control clones. The great diversity of the levels of *Adam17* silencing in individual clones may be explained by the aneuploidy of MC38CEA cells and the appearance of indel-containing transcript molecules prior to their destruction via nonsense-mediated decay (NMD).

Remarkably, the levels of *Nos2* transcripts were in the range of 20–270% of the mean value calculated for control clones and there was no correlation between the expression of *Adam17* and *Nos2* (Fig. 1E; Fig. S4). We also compared the results of RNAseq analyses of unstimulated and cytokine-stimulated MC38CEA cells, in which ADAM17 expression was silenced by shRNAs or CRISPR/Cas9 (GEO repository accession numbers: GSE181084 and GSE181271, respectively) (Czarnek and Bereta, 2021a,b). The analysis did not reveal common inflammation-related genes affected by diminished ADAM17 expression, independently of the method of its silencing.

The finding that A17.2 and A17.5 shRNAs designed to target *Adam17* transcript diminished also ADAM10 expression prompt us to form a hypothesis that there is a correlation between expression of ADAM10 and that of iNOS. Indeed, three commercial shRNA sequences targeting ADAM10 resulted in diminished expression of *Nos2* in MC38CEA cells (Fig. 1A,B). That A17.3 shRNA resulted in slight increase in both *Adam10* and *Nos2* expression further supported our belief of dependence of the level of *Nos2* expression on that of *Adam10* (Fig. 1A,B).

The effects of A17.5 and A10.1 shRNAs were moderate and the efficiency of silencing of their targets decreased over time, therefore we limited further studies to MC38CEA cells expressing three shRNAs that diminished *Adam10* and *Nos2* expression, namely A17.2, A10.2, and A10.3, and one, A17.3 that caused slight increase in expression of both *Adam10* and *Nos2*.

The levels of *Nos2* pre-mRNA and mature mRNA were similarly affected by specific shRNAs (Fig. 1F) and shRNA-mediated inhibition was detectable after the shortest stimulation time tested, i.e. in 2 h after inducing *Nos2* transcription with IL1β and IFNγ (Fig. S5). This indicated that the changes in *Nos2* mRNA levels resulted from the regulation of *Nos2* transcription rather than from the changes in transcript stability, which could occur in the case of its direct interaction with shRNA-derived siRNAs. We confirmed this notion in a single experiment in which we have measured *Nos2* transcript levels after inhibition of transcription by alpha-amanitin. After 6 h of the alpha-amanitin exposure the levels of *Nos2* mRNA in MC38CEA cells expressing control or A17.2 shRNA decreased respectively to 92.5% and 95% of its initial value. Although efficient stimulation of *Nos2* transcription in MC38CEA cells requires two inducers, *Nos2* mRNA is moderately increased in response to a single stimulant. *Adam10*-specific shRNAs decreased the levels of IL1β-induced- as well as IFNγ-induced *Nos2* expression (Fig. S5).

We applied a luciferase reporter assay to analyze the effect of the shRNAs on activity of *Nos2* promoter. The luciferase coding sequence was under control of the 1.75 kb fragment derived from a promoter and first exon of *Nos2.* The shRNAs influenced both basal and cytokine-stimulated activity of *Nos2* promoter (Fig. 1G). The pattern of changes in the levels of luciferase under the control of the *Nos2* promoter was consistent with the levels of endogenous *Nos2* in the cell lines with silenced ADAM17 and/or ADAM10 expression.

**Fig. 2.**
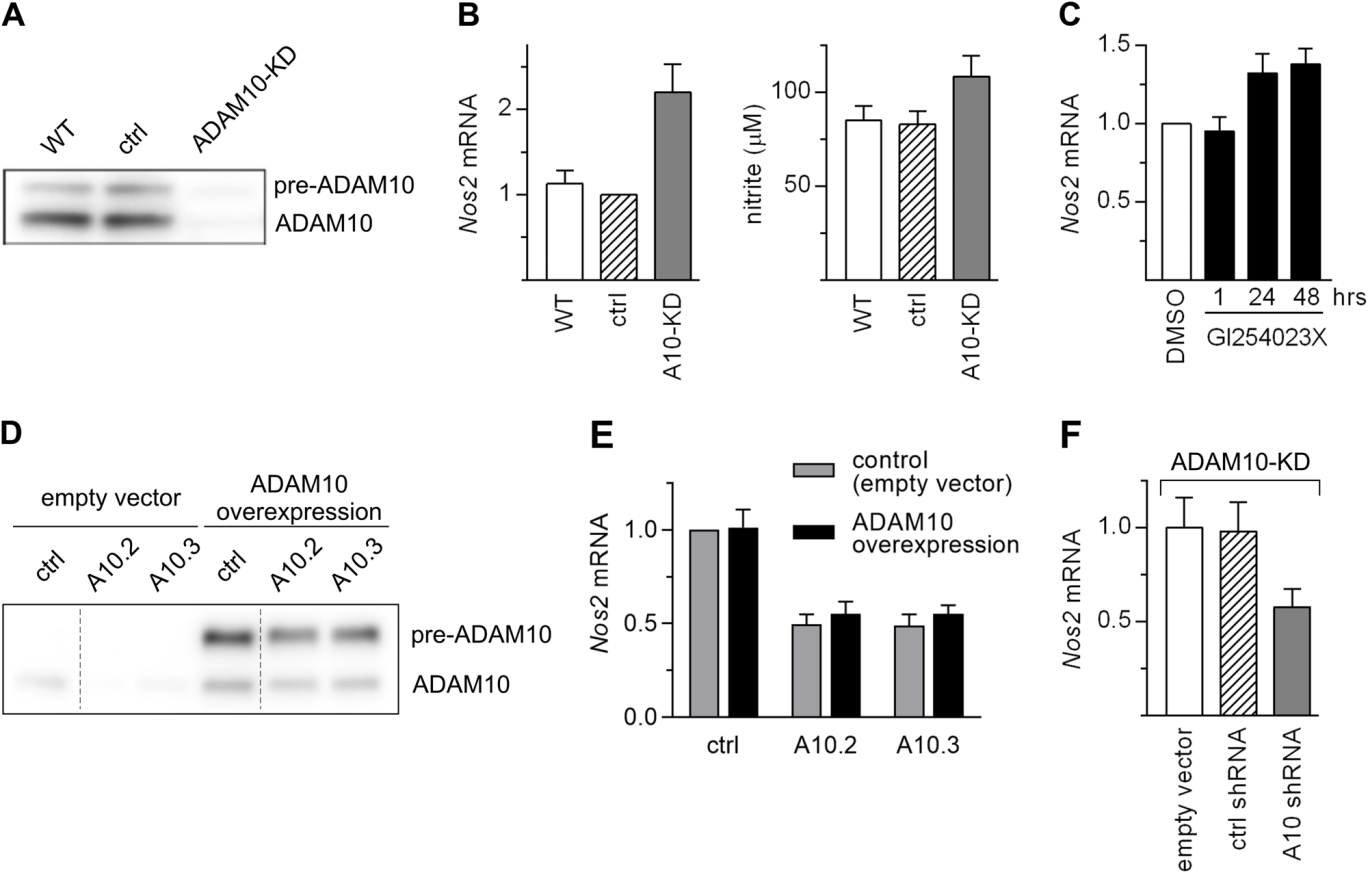
**Diminished ADAM10 expression due to CRISPR/Cas9 gene editing did not correlate with reduced inducibility of *Nos2*.** (A) WB analysis of ADAM10 levels in MC38CEA cells, in which *Adam10* expression was knocked down by CRISPR/Cas9. In control cells *EGFP*-specific sgRNA was used instead of *Adam10*-specific sgRNAs. Representative WB image of two independent experiments is presented. Whole WB and Ponceau S-stained membranes are given in Fig. S7 (B) RT-qPCR analysis of *Nos2* mRNA levels and measurement of nitrite levels in lysates and media, respectively, of MC38CEA cells described in (A), stimulated with IL1β and IFNγ for 6 h (RT-qPCR) or 20 h (nitrite). Data are shown as MV±s.d. from three (qPCR) or four (nitrite levels measurement) independent experiments. (C) RT-qPCR analysis of *Nos2* mRNA levels in MC38CEA cells incubated for indicated times with ADAM10 inhibitor, GI254023X (10 µM) or with its solvent, DMSO (for 48 h) before addition of cytokines for the next 6 h. Data are shown as MV±s.d. from three independent experiments. (D) WB analysis of the levels of ADAM10. MC38CEA cells expressing non-targeting-, or A10.2-, or A10.3 shRNA were transfected with an empty vector or a vector encoding shRNA-resistant version of *Adam10*. After antibiotic selection the cells were lysed and proteins were analyzed using WB. Representative WB image of two independent experiments is shown. For clarity of presentation two lanes were removed from the image (the sites are indicated by vertical lines). Whole WB and Ponceau S-stained membranes are given in Fig. S7. (E) RT-qPCR analysis of *Nos2* mRNA levels in MC38CEA cells transduced with vectors silencing ADAM10 (10.2 and 10.3) or non-targeting shRNA (ctrl), and then transfected with vector coding for ADAM10 or empty vector, stimulated for 6 h with IL1β and IFNγ. (F) RT-qPCR analysis of *Nos2* mRNA levels in *Adam10*-KD MC38CEA transduced with an empty vector or vector encoding non-targeting shRNA (ctrl), or *Adam10* shRNA. RNA was isolated from the cells stimulated for 6 h with IL1β and IFNγ.

To verify the notion that the levels of ADAM10 influence the expression of iNOS we knocked down *Adam10* expression using CRISPR/Cas9 gene editing in both MC38CEA and P388D1 cells (Fig. 2A; Fig. S6).

**Fig. 3.**
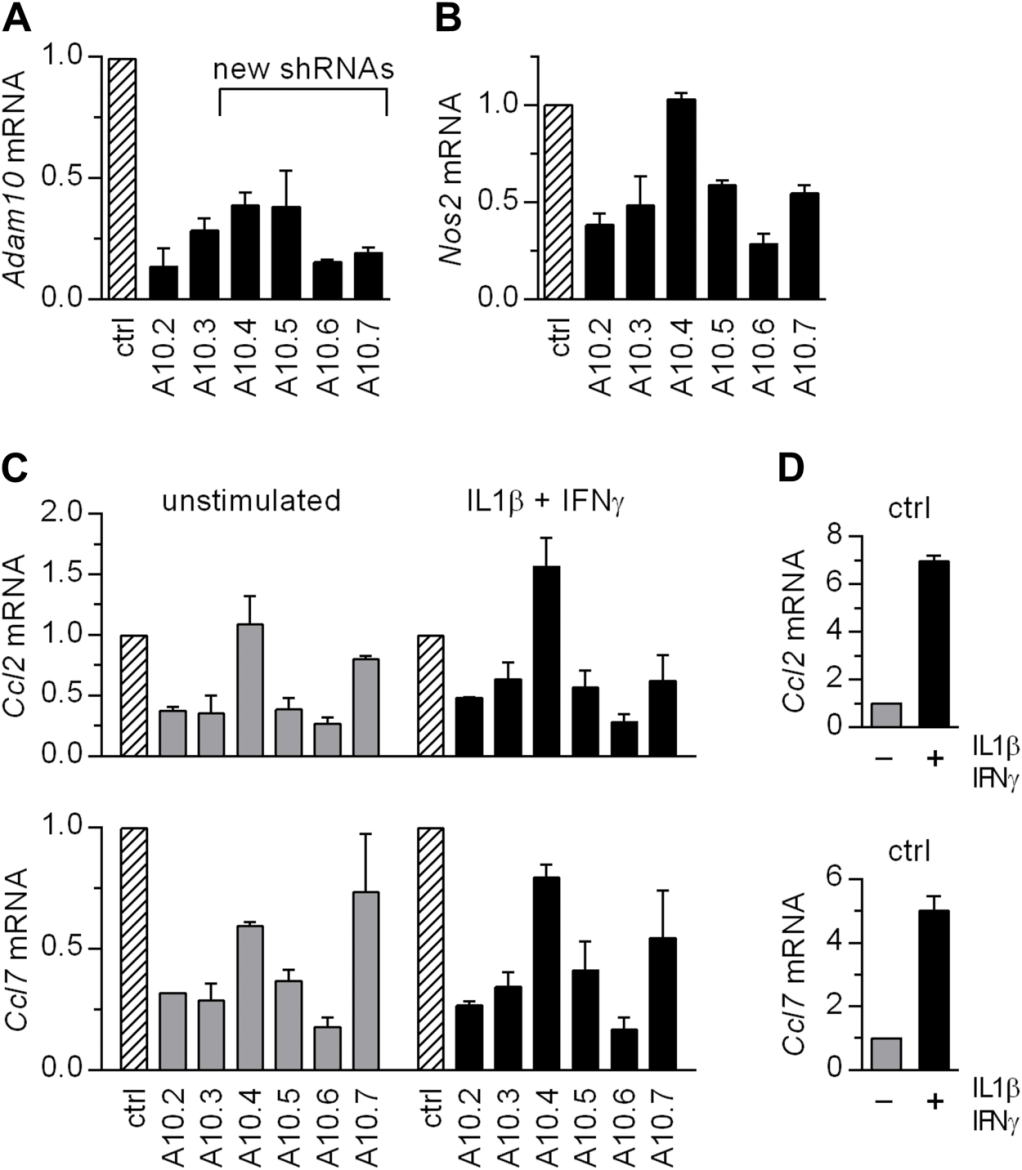
**Majority of shRNAs targeting *Adam10* diminished levels of proinflammatory transcripts.** RT-qPCR analysis of (A) *Adam10*, (B) *Nos2*, and (C) *Ccl2* and *Ccl7* mRNA levels in MC38CEA cells expressing not-targeting shRNA or one of *Adam10* shRNAs. The cells were left untreated or were stimulated with IL1β and IFNγ for 6 h prior to RNA isolation. The relative levels of the transcripts in control cells (transduced with vector encoding non-targeting shRNA) were taken as 1. (D) Magnitude of cytokine stimulation of *Ccl2* and *Ccl7* mRNAs in control cells. Data are presented as MV±s.d. from two independent experiments.

Surprisingly, although the level of ADAM10 in *Adam10-*KD MC38CEA cells was strongly diminished, the levels of *Nos2* expression and iNOS activity were increased rather than decreased (Fig. 2B). A specific inhibitor of ADAM10 proteolytic activity, GI254023X, also slightly stimulated *Nos2* expression in MC38CEA cells (Fig. 2C). Knocking-down ADAM10 expression in P388D1 also did not inhibit iNOS expression and activity (Fig. S6). These results suggested that certain shRNAs targeting *Adam10*, but not ADAM10 silencing by itself, influence iNOS expression. To confirm this hypothesis, we introduced silent mutations to the sites within *Adam10* coding sequence targeted by A10.2 and A10.3 in the ADAM10-encoding vector to make it resistant to shRNA-mediated degradation (Fig. 2D). Overexpression of shRNA-resistant version of ADAM10 in MC38CEA cells expressing A10.2- or A10.3 shRNA did not restore the normal levels of *Nos2* transcript in the cells stimulated with IL1β and IFNγ (Fig. 2E). Moreover, when *Adam10*-KD MC38CEA cells, which produce negligible amounts of ADAM10 (Fig. 2A), were transduced with the vector encoding A10.3 shRNA, the expression of *Nos2* was diminished (Fig. 2F). These results confirmed the lack of correlation between the levels of ADAM10 and inducibility of *Nos2*. We generated four additional vectors encoding shRNAs targeting ADAM10, named A10.4, A10.5, A10.6, and A10.7 to verify the existence of a link between expression of such shRNAs and iNOS expression. All of them reduced the levels of *Adam10* by 60–80% (Fig. 3A), and all of them but A10.4 resulted in diminished levels of cytokine-induced *Nos2* expression (Fig. 3B). The studied shRNAs did not markedly stimulate expression of the interferon-inducible genes, *Oas1b* and *Ifit1* (Fig. S8). Thus, their effects cannot be attributed to the induction of interferon response, which might be triggered by some shRNAs and might affect cell viability and homeostasis.

MC38CEA cells express only few proinflammatory proteins (GEO repository: GSE181084 or GSE181271) (Czarnek and Bereta, 2021a,b). We chose CCL2 (C-C chemokine ligand 2), known also as MCP1 (macrophage chemoattractant protein 1) and CCL7 (MCP3) to examine whether the influence of shRNAs was limited to iNOS expression or is a more general phenomenon. The expression of *Ccl2* and *Ccl7* was affected by specific shRNAs in the same way as that of *Nos2* (Fig. 3C). The data indicated that the impact of specific shRNAs on expression of inflammatory genes might involve a common mechanism. Unlike *Nos2*, which is undetectable in the absence of inflammatory stimuli, *Ccl2* and *Ccl7* are expressed at low levels in unstimulated cells. shRNAs affected also this basal *Ccl2* and *Ccl7* expression (Fig. 3C).

The expression of *Ccl2* and *Ccl7* was also affected in a similar way as that of *Nos2* by A17.2 and A17.3 shRNAs (Fig. S9).

The expression of all analyzed proinflammatory genes: *Nos2*, *Ccl2*, and *Ccl7* is regulated by NF-κB, AP-1, and STAT1 transcription factors (Deng et al., 2013; Tang et al., 2020; Thompson and Van Eldik, 2009; Zhang et al., 2020). We decided to evaluate whether studied shRNAs influence their activation. We encountered a problem of the gradually decreasing degree of *Adams* silencing, resulting probably from a progressive decline in the average number of lentiviral cassettes per cell in the cell cultures. This could have influenced the reproducibility of the results as other effects of shRNAs could have been also diminished. To address this issue and to ensure that the effects of transduction/selection on the transcription factors would be temporally separated from these of shRNAs we generated vectors with the tetracycline/doxycycline-inducible expression of shRNAs. We chose four shRNAs: A17.2, A17.3, A10.2, and A10.3.

**Fig. 4.**
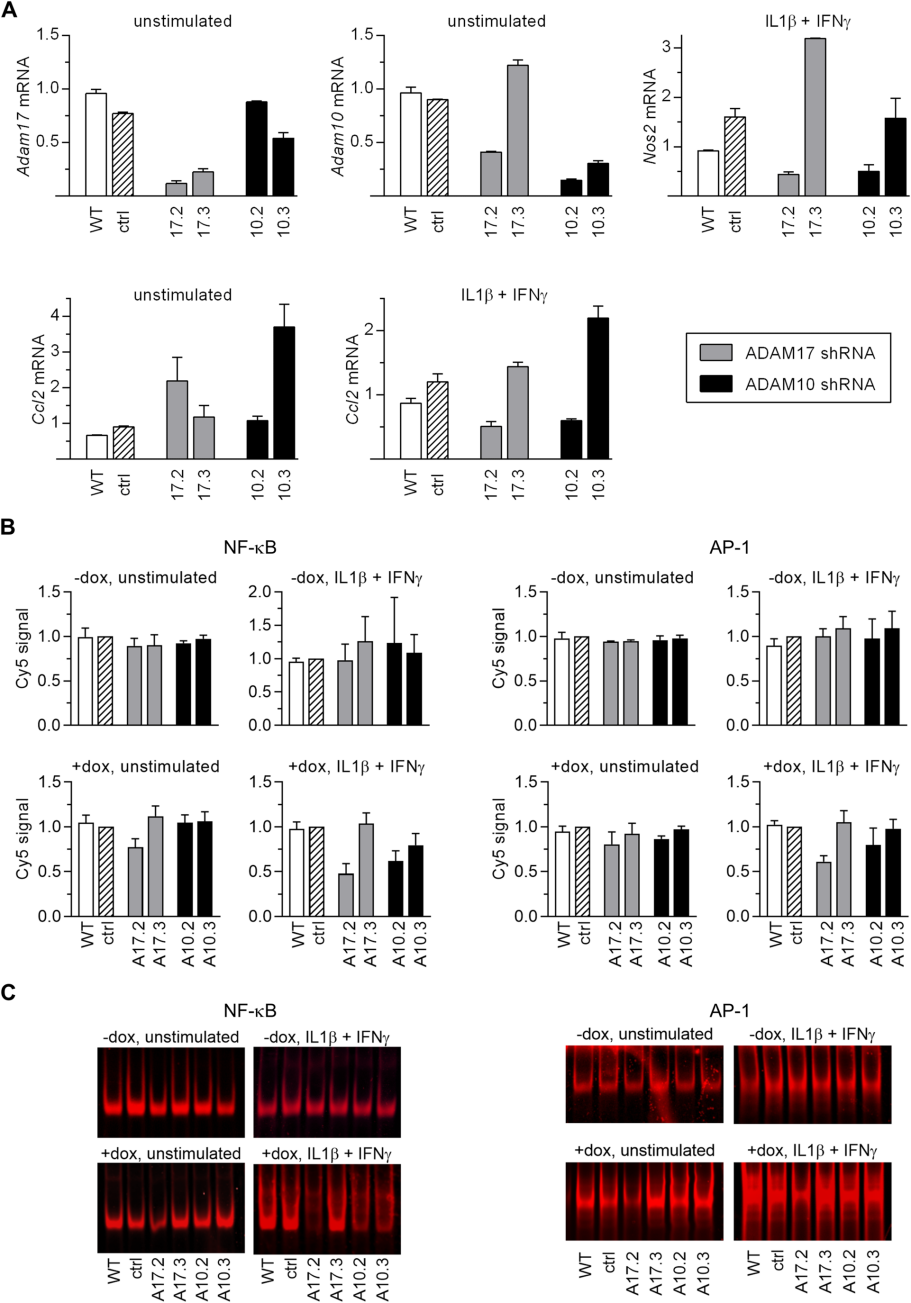
**Doxycycline-induced shRNAs influence expression of proinflammatory genes.** (A) RT-qPCR analysis of mRNA levels of *Adam17*, *Adam10*, *Nos2*, and *Ccl2* in WT MC38CEA cells or MC38CEA transduced with doxycycline-inducible vectors encoding not-targeting shRNA (control, ctrl) or one of analyzed shRNAs. Expression of shRNAs was switched on with doxycycline (200 ng/ml) 3 days prior to RNA isolation. The cells were left unstimulated or were stimulated with IL1β and IFNγ for the last 6 h of experiment. Data are presented as a ratio of mRNA levels in dox-induced cells to that in uninduced cells. Data are presented as MV±s.d. from two independent experiments. (B) Analysis of NF-κB and AP-1 activation in MC38CEA cells described in A by EMSA. The cells were left untreated or were stimulated with IL1β and IFNγ for 30 min prior to isolation of nuclear proteins. For all experimental groups MV±s.d. from at least three independent experiments are shown; only for AP-1 analysis in the cells untreated with doxycycline and unstimulated data are from two experiments. (C) Exemplary images of EMSA. Uncropped images are presented in Fig. S11.

A17.2, A17.3, and A10.2 induced with doxycycline had the same effect on *Adam17* and *Adam10* levels and on *Nos2* expression as their constitutively expressed counterparts (Figs 4A and 1A). However, constitutive and inducible expression of A10.3 shRNA elicited different responses in terms of *Nos2* expression after cytokine stimulation. Although in both cases the levels of *Adam10* was decreased, *Nos2* expression was not diminished in response to inducible expression of A10.3 shRNA (Figs 4A and 1A). It is possible that shRNAs transcribed from different vectors are differently processed and the products might have overlapping but not identical activities (Putzbach et al., 2017).

Similarly, as in the case of constitutively expressed shRNAs, the pattern of effects of specific dox-induced shRNAs on cytokine-stimulated expression of *Ccl2* resembled that of *Nos2* (Fig. 4A). The data confirmed that the impact of specific shRNAs on expression of inflammatory genes did not depend on ADAMs expression levels. The effects of all analyzed shRNAs, either expressed constitutively or induced by dox are summarized in Table S2.

We next evaluated the influence of dox-induced expression of studied shRNAs on the activation of NF-κB, AP-1, and STAT1 transcription factors involved in the regulation of *Nos2*, *Ccl2* and *Ccl7* expression. We included also Sp1 transcription factor essential for expression of *Ccl2* (Ping et al., 2000).

The activity of NF-κB was decreased in unstimulated cells expressing A17.2 shRNA by ∼20%. In cytokine-stimulated cells expressing A17.2 shRNA NF-κB activity was reduced by ∼50%, and in those expressing A10.2 shRNA by ∼40% compared to control cells, i.e. cytokine-stimulated cells expressing non-targeting shRNA. These data point to NF-κB as a factor most significantly influenced by shRNAs. This conclusion is not fully supported by our experiments performed with the cells with constitutive expression of shRNAs. Removal of NF-κB sequences from *Nos2* promoter abolished the stimulation of luciferase expression by cytokines but did not abolish the effect of shRNAs on the basal level of its expression (Fig. S10). This indicates the existence of other than NF-κB elements affected by shRNAs required for efficient activation of *Nos2* promoter. The EMSA analysis revealed that dox-inducible shRNAs also influenced activity of AP-1 although to a lesser extent than that of NF-κB. Expression of A17.2 shRNA decreased AP-1 activity in cytokine-stimulated cells by ∼40% and of A10.2 shRNA by ∼20% compared with control cells. A17.3 and A10.3 shRNAs did not substantially affect NF-κB and AP-1 activities. The binding of STAT and Sp1 to their consensus sequences were not influenced by any of the analyzed shRNAs (Fig. S12).

## DISCUSSION

The results of our work highlight a number of issues that must be taken into account in gene silencing experiments to avoid false conclusions. One threat comes from establishing cell lines with silenced expression of a gene of interest from clones of a single cell. The necessity to use the clones is a result of inefficient gene transfer to the cells transfected with plasmids encoding shRNAs or inefficient knockdown of a gene by CRISPR/Cas9. We observed tremendous variability in the levels of *Nos2* expression in the clones of MC38CEA cells, in which *Adam17* was knocked down with CRISPR/Cas9. Remarkable heterogeneity of cells, including cell transcriptomes, in cultures of established cell lines is a well-recognized phenomenon (Hu et al., 2019; Rajaram et al., 2017). It is therefore possible to select quite randomly several clones, in which silencing of a given gene is accompanied by the same feature. Moreover, silencing of a specific gene may promote growth of cells with certain characteristics. High or low expression of certain genes may mitigate the adverse effects associated with silencing of a specific gene. In this scenario, the two features occur in parallel, but there is no cause-and-effect relationship between them. We believe that our original observation of a decrease in *Nos2* expression in *Adam17*-silenced clone-derived cell lines (Fig. S1) might be accidental or resulted from off-target effects of the used shRNA. In the present work we ruled out the correlation between *Adam17* levels and cytokine-induced *Nos2* expression in MC38CEA cells.

The second threat is a lack of stringent specificity of shRNAs, which may lead to silencing of closely related genes. In our studies, none of the sequences targeting *Adam10* silenced *Adam17* expression, but two of the sequences targeting *Adam17* diminished the expression of *Adam10*. If these sequences were used to distinguish the activity of ADAM17 from that of ADAM10, it could result in false conclusions. Unintended silencing of related genes is part of a larger phenomenon known as off-target effects. Bioinformatic tools are used to design shRNAs, but taking into account the current state of knowledge, they cannot ensure specificity of indicated sequences. The potential off-targets of a given shRNA remain difficult to predict because of the alternative selection of siRNA strands, lower complementarity requirements than were initially assumed, and variations in shRNA-derived siRNAs.

Argonaute protein (Ago) is responsible for a choice of a guide strand of an miRNA or siRNA duplex. It supposedly favors the strand, which has a 5′ terminus in the less thermodynamically stable end of the duplex and U as the first nucleotide. However, half of human miRNAs elude one or both of these rules (Medley et al., 2020). Likewise, the strand of shRNA designed to be a passenger strand may play a role of a guide strand (Sheng et al., 2020), thus increasing the number of potential off-targets. Although shRNAs are designed to have ∼20 nucleotides complementary to specific targets, the complementarity of a six nucleotide-seed sequence may be sufficient for off-target activity (Birmingham et al., 2006; Gao et al., 2018; Murmann et al., 2020). Other profiles of complementarity between miRNA and mRNA may also provide effective silencing of certain transcripts (Akbari Moqadam et al., 2013; Brennecke et al., 2005). Additionally, one pre-miRNA may be processed into isomiR variants, which slightly differ in length and/or the terminal nucleotide(s) (Gebert and MacRae, 2019). Small differences in processing of shRNAs encoded by different vectors were also reported (Putzbach et al., 2017). Also, certain shRNAs encoded by TRC vector, thus possessing the same hairpin scaffold as commercial *Adam*-targeting vectors used in our studies, were shown to be imprecisely processed (Gu et al., 2012; Watanabe et al., 2016) or processed via alternative, Dicer-independent mechanism generating a set of various siRNA, which targeted unintended transcripts (Bhinder et al., 2014). We believe that the differences in off-target activities of constitutively-expressed versus dox-inducible A10.3 shRNA might be due to the fact that the transcribed molecules may slightly differ in 5′-terminus and thus undergo non-identical processing.

The most impressive example of serious consequences of off-target effects comes from works of Lin et al. (Lin et al., 2019; Lin et al., 2017). They demonstrated how erroneous RNAi data led to selection of incorrect targets for cancer therapy and even to initiation of clinical trials, which were bound to fail. In our previous work we reported that also control shRNA, which by definition should not influence expression of any gene, might elicit powerful off-target effects that invalidate the reliable interpretation of RNAi data (Czarnek et al., 2021). Thus, in the case of shRNAs application, off-target effects seem to be a common phenomenon. To minimize the likelihood of incorrect conclusions, several different shRNAs targeting the same transcript are usually used, because each shRNA is assumed to have a distinct set of off-targets. The third threat arises from the belief that this approach solves the problem; that if several different shRNAs targeting a given transcript led to the same accompanying effect, then this effect is a consequence of the gene silencing. In our work the expression of eight shRNAs out of nine that diminished *Adam10* levels (seven designed to target *Adam10* and two designed to target *Adam17*) resulted in a decrease of cytokine-induced transcription of *Nos2* in MC38CEA cells. Contrary to expectations, ADAM10 silencing was not responsible for this effect, which we demonstrated using a wide variety of experimental approaches (Fig. 2).

This observation of alike off-target effects revealed by a set of shRNAs targeting the same mRNA is not absolutely unique. Putzbach et al. found that more than 80% of numerous si/shRNA targeting CD95 and CD95L induced cell death through unexpected targeting of a number of critical survival genes (Putzbach et al., 2018, 2017). Further studies by the same group revealed that G-rich hexamer seed sequences, including those derived from CD95L transcript, are required and sufficient for targeting C-rich 3′UTR of survival genes (Gao et al., 2018). The authors propose that the phenomenon reflects the simultaneous evolution of tumor suppressor miRNAs and 3'UTRs of majority of mRNAs: the increase in G abundance in a guide strand of miRNAs was accompanied by the decrease of Cs in most mRNAs but not in survival gene transcripts (Gao et al., 2018).

The shRNAs targeting *Adam10* affect expression of a set of pro-inflammatory genes and, as we showed for two shRNAs, decrease activity of two transcription factors, NF-κB and AP-1. We believe that analyzed shRNAs share a common trait which determines their anti-inflammatory properties. The complex bioinformatic analysis of 3′UTRs of transcripts coding for proinflammatory proteins is not yet available. Therefore, the idea that by analogy with the CD95L shRNAs targeting survival genes, shRNAs specific towards *Adams* target unintended group of proinflammatory genes, while tempting, is highly speculative.

Another possibility is that these shRNAs target one or a few molecules that play a central role in the proinflammatory activation of the cells. For example, they might interfere with the complex net of miRNAs and lncRNAs involved in the regulation of inflammation and immune responses (Mahesh and Biswas, 2019; Marques-Rocha et al., 2015; Testa et al., 2017). They could also affect mRNAs coding for proteins that potentiate pro-inflammatory signaling pathways as, e.g. various kinases or poly(ADP-ribose) polymerase 1 (PARP-1) (Bai and Virag, 2012; Ke et al., 2019) or ubiquitin-specific protease 14 (USP14) (Liu et al., 2017). Also, more complex or diverse or overlapping scenarios for different shRNAs are possible. The elucidation of the mechanism of off-target effects of shRNA targeting *Adam10* is beyond the scope of this study.

In recent years CRISPR/Cas9-based methods (CRISPRko and CRISPRi) evolved as an alternative for RNAi (Housden and Perrimon, 2016; Schuster et al., 2019). Although CRISPR/Cas9-based methods are not flawless, they outcompete RNAi in terms of minimizing off-target effects (Doench et al., 2016; Evers et al., 2016; Naeem et al., 2020). In the case of CRISPRi, in which Cas9 is not enzymatically active and the efficient inhibition of transcription requires long-term and high-affinity interaction between 17–20 nucleotide guide sequence and target DNA, the number of off-targets is negligible (Gilbert et al., 2014; Peddle et al., 2020). We have successfully applied both CRISPRko (Fig. 2; Fig. S6) and CRISPRi methods (manuscript in preparation) to efficiently inhibit *ADAM10* expression and verify the results of shRNA-based experiments.

Our work stresses the necessity of providing strict controls in RNAi experiments including restoration of normal levels of a studied protein, which should reverse the effects of the silencing of its expression. It would also be reasonable to verify RNAi results with complementary methods, e.g. CRISPR/Cas9-based techniques.

## MATERIALS AND METHODS

### Cell culture

MC38CEA (murine colon adenocarcinoma cell line (Corbett et al., 1975) expressing human carcinoembryonic antigen (Bereta et al., 2007) and P388D1 (mouse monocyte/macrophage-like cell line, ATCC^®^ CCL-46) were grown in DMEM High Glucose (BioWest) supplemented with 5% heat-inactivated fetal bovine serum (FBS, BioWest) at standard conditions. The cells transduced with doxycycline-inducible shRNA expression vectors were cultured in the presence of heat-inactivated, tetracycline-free FBS (BioWest). The cells were passaged by trypsinization (MC38CEA) or by dislodging with a sterile cell scraper (P388D1) after reaching 80–90% confluence. The cells were tested for *Mycoplasma* contamination using PCR (primer sequences are given in Table S3) and cultured in the absence of preventive antibiotics, penicillin and streptomycin.

### shRNA constructs, lentiviral vector production and transduction

Lentiviral vectors from MISSION^®^ library encoding non-targeting shRNA (SHC002) and mouse ADAM17- and ADAM10 shRNAs were purchased from Sigma-Aldrich (presently Merck). Additional vectors containing ADAM10 shRNAs under constitutive hU6 promoter and vectors with doxycycline-inducible shRNAs were produced in-house. pLKO.1-puro (Stewart et al., 2003) (a gift from Bob Weinberg, Addgene plasmid #8453) or Tet-pLKO-puro (Wiederschain et al., 2009) (a gift from Dmitri Wiederschain, Addgene plasmid #21915) were digested with *Age*I and *Eco*RI, resolved in an agarose gel and extracted using Gel-Out Concentrator (A&A Biotechnology). Oligonucleotides encoding the shRNA sequences (Table S1) were annealed in 1×Taq buffer and ligated into linearized plasmids. Lentiviral vectors were produced as described previously (Czarnek et al., 2021).

The cells were plated in 12-well plates at a density of 125,000 cells per well. The next day, cells were transduced with equal volumes of concentrated media containing pseudoviruses (the optimal volume was determined experimentally by transducing cells with different volumes of a concentrated viral stock). The volumes that yielded about 50–80% of puromycin-resistant cells were eventually used for transduction of both cell lines via spinoculation at 1150 ***g*** for 30 min at room temperature in the presence of polybrene (8 µg/ml). After 2 days, puromycin was added to the cultures at a concentration of 5 µg/ml for MC38CEA cells and 1 µg/ml for P388D1 cells.

### Cell stimulation

Recombinant human interleukin 1β (IL1β) and recombinant mouse interferon γ (IFNγ) were from BioLegend. Both cytokines were used at a concentration of 10 ng/ml. *Escherichia coli* lipopolysaccharide serotype 0111:B4 (LPS, Sigma-Aldrich) was used at a concentration of 100 ng/ml.

### RNA isolation, reverse transcription and RT-qPCR

The cells were seeded at 125,000 cells per well onto 12-well plates 2 days prior to stimulation. The cells were stimulated with IL1β and IFNγ (MC38CEA) or LPS (P388D1) for 6 h. RNA was isolated using phenol/chloroform extraction with Fenozol reagent (A&A Biotechnology) as recommended by the manufacturer. pH of the Fenozol was adjusted to 4.5 with acetic acid before use. If pre-mRNA was to be analyzed, residual DNA was removed by digestion with TURBO DNase (Thermo Fisher Scientific) for 45 min at 37°C and RNA was further purified using Clean Up RNA Concentrator (A&A Biotechnology). RNA concentration was determined spectrophotometrically using NanoDrop ND-1000 (NanoDrop Technologies LLC). Equal amounts of RNA (1 μg) were reverse-transcribed with M-MLV polymerase (Promega) and oligo(dT)_15_ (or a mixture of oligo(dT)_15_ and random hexamer primers for pre-mRNA analysis). RT-qPCR reactions were performed using AceQ qPCR SYBR Green Mix (Vazyme Biotech) on Eco Real-Time PCR System (Illumina). The levels of analyzed transcripts were normalized to a geometric mean of two reference genes: *Eef2* and *Polr2b*. Primers for mRNA analysis were designed to span different exons with a minimal intron size of 1000 nt; primers for pre-mRNA analysis were designed to bind within exon and adjacent intron; alternatively, both primes were specific for intronic sequences. The sequences of all primers are listed in Table S3.

### Nitrite concentration measurements – Griess assay

The cells were seeded at 10,000 cells per well in 96-well plates 2 days prior to stimulation. The next day they were stimulated with IL1β and IFNγ (MC38CEA) or LPS (P388D1) for 20 h. The collected media (100 μl) were mixed with equal volume of freshly prepared Griess reagent (1:1 mixture of 0.5% sulfanilamide and 0.05% *N*-(1-Naphthyl)ethylenediamine, both in 5% phosphoric acid) and incubated at room temperature for 5 min. The absorbance at 545 nm was measured using a microplate reader Synergy H1 Hybrid (BioTek). Nitrite concentration was determined using standard curve of known concentrations of sodium nitrite in culture medium.

### Western blotting

The cells were lysed in ice-cold RIPA buffer enriched with 5 mM EDTA, Halt Protease Inhibitor Cocktail (Thermo Fisher Scientific), and 5 μM ADAM10-specific inhibitor GI254023X (Brummer et al., 2018). After brief sonication, protein samples (25 μg) were subjected to tris-glycine SDS-PAGE and transferred onto 0.45 μm PVDF membranes (Immobilon P, Merck). The membranes were stained with Ponceau S to ensure equal protein loading and images were taken. The membranes were destained, blocked in 5% non-fat dried milk in TBST and probed with rabbit-anti ADAM17 at 1:2500 (PA5-17080, Thermo Fisher Scientific) or rabbit anti-ADAM10 at 1:5000 (ab1997, Abcam) and then with HRP-conjugated secondary antibody (goat anti-rabbit at 1:10,000, Sigma-Aldrich). Bands were developed with Immobilon Western Chemiluminescent HRP Substrate (Merck) and visualized using Fusion FX (Vilber Lourmat). The exposition time was set to ‘auto’.

### Generation of *Adam*-knockdown cell lines with Cas9-mediated genome editing

Generation of *Adam17*-KD cells was described previously (Karabasz et al., 2021). *Adam10-*KD cells were generated similarly with minor modifications. Briefly, Cas9 coding sequence was PCR-amplified from pX330-U6-Chimeric_BB-CBh-hSpCas9 (Cong et al., 2013) (hereinafter referred to as pX330; a gift from Feng Zhang, Addgene plasmid #42230) and cloned into pJET1.2-blunt (Thermo Fisher Scientific). The plasmid with the insert oriented such that Cas9 CDS was located downstream of the T7 promoter was purified using Plasmid Midi AX (A&A Biotechnology), linearized with *Xba*I and DNA was phenol/chloroform-extracted. Polyadenylated, capped mRNA was produced using HiScribe T7 ARCA mRNA Kit with tailing (New England Biolabs) and purified by LiCl precipitation. sgRNA sequences targeting sequences within exon 2, 3, 4, and 5 of mouse *Adam10* gene were designed using GPP sgRNA Designer (Broad Institute). sgRNA targeting EGFP was used as a control. Sequences of sgRNAs are available in Table S4. Oligonucleotides encoding sgRNAs targeting *Adam10* or *EGFP* were annealed in 1×Taq buffer and cloned into pX330 in restriction-ligation reaction containing the vector, annealed oligonucleotides, FastDigest BpiI (Thermo Fisher Scientific) and T4 DNA ligase (Thermo Fisher Scientific) in 1×T4 DNA ligase buffer. sgRNAs were PCR-amplified with primers containing T7 promoter sequence and purified PCR products were transcribed using TranscriptAid T7 High Yield Transcription Kit (Thermo Fisher Scientific). *In vitro* transcribed sgRNAs were purified using LiCl precipitation.

The cells were detached from culture vessels by trypsinization (MC38CEA) or by scraping (P388D1), washed with electroporation buffer (EB; 100 mM sodium phosphate, 50 mM sodium succinate, 20 mM HEPES, 10 mM MgCl_2_, 5 mM KCl, pH 7.2) and electroporated in EB with 2 μg of Cas9 mRNA using Gene Pulser II (Bio-Rad) in 0.4 cm gap cuvettes (Bio-Rad) with following pulse parameters: 400 V, 500 μF (MC38CEA) or 500 V, 500 μF (P388D1). The procedure was repeated after 4 h, but with 1 µg of sgRNA targeting *Adam17*, *or Adam10*, or *EGFP* instead of Cas9 mRNA. The cells were allowed to regenerate in culture (5–7 days), and the electroporation was repeated with another sgRNA (two in total).

### Construction of a plasmid coding for ADAM10 resistant to shRNA

ADAM10 coding sequence was PCR-amplified from LeGO-ADAM10 (Czarnek and Bereta, 2020) with primers containing *Sfi*I sites, digested overnight with *Sfi*I and cloned into *Sfi*I-digested pSBbi-Bla (Kowarz et al., 2015) (a gift from Eric Kowartz, Addgene plasmid #60526). A fragment of ADAM10-coding sequence that contains sites targeted by *Adam10*-specific shRNA sequences 2 and 3 was codon-optimized to retain correct amino acid sequence but abolish shRNA-mediated mRNA degradation. dsDNA was synthetized (GeneArt Strings DNA Fragments, Thermo Fisher Scientific) and ligated into PCR-amplified pSBbi-Bla containing the remaining portion of ADAM10 CDS using NEBuilder HiFi DNA Assembly Cloning Kit (New England Biolabs).

MC38CEA cells were seeded in 12-well plates one day prior to transfection. The cells were transfected with 950 ng of pSbbi-Bla containing shRNA-resistant version of ADAM10 or an empty plasmid together with 50 ng of SB100X transposase-encoding vector pCMV(CAT)T7-SB100 (Mates et al., 2009) (a gift from Zsuzsanna Izsvak, Addgene plasmid #34879) using jetPRIME transfection reagent (Polyplus Transfection) following the manufacturer's recommendations. After 24 h, the medium was replaced with the fresh one containing blasticidin S (8 μg/ml).

### Reporter constructs, transfection and luciferase reporter assay

pGL2-basic, a promoterless plasmid containing the firefly luciferase coding sequence, was purchased from Promega. pGL2-NOS2Promoter-Luciferase (Lowenstein et al., 1993) (hereinafter referred to as pGL2-NOS2prom) was a gift from Charles Lowenstein (Addgene plasmid #19296). pGL2-NOS2promΔNFκB containing a version of *Nos2* promoter lacking two NF-κB binding sites was generated using Gibson assembly method. A fragment between NF-κB binding sites and the pGL2-NOS2prom (excluding NF-κB binding sites) were PCR-amplified and ligated using NEBuilder HiFi DNA Assembly Cloning Kit (New England Biolabs). MC38CEA cells were seeded at 8000 cells/well in 96-well white plates. The next day the cells were transfected in duplicates with 95 ng of one of the firefly luciferase-encoding plasmids together with 5 ng of plasmid coding for *Renilla* luciferase under constitutive SV40 promoter using jetPRIME (Polyplus Transfection). The next day the medium was replaced with the fresh one and after further 24 h the cells were stimulated with IL1β and IFNγ for 6 h. After this time luciferase levels were determined using Dual-Glo Luciferase Assay System according to the manufacturer's protocol. The luminescent signals were measured using a microplate reader Synergy H1 Hybrid (BioTek) and calculated for each sample following the equation:

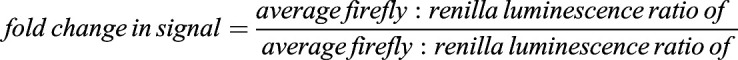


The signal of unstimulated cells transfected with control shRNA were taken as 1.

### Electrophoretic mobility-shift assay (EMSA)

MC38CEA cells carrying doxycycline-inducible vectors coding for control or ADAM-specific shRNAs were cultured in plates (Ø=6 cm). The cells were treated for 72 h with doxycycline and then for 30 min with IL1β and IFNγ. Nuclear extracts were prepared by the method of Suzuki et al. (Suzuki et al., 1994) with slight modifications. In brief, ∼3×10^6^ cells at a confluence of ∼80%, were washed with PBS, harvested, and incubated on ice for 15 min in 10 mM Tris-HCl buffer pH 7.5 containing 10 mM NaCl, 3 mM MgCl_2_, 1 mM DTT, and 0.2 mM PMSF. Nonidet NP-40 was added to a final concentration of 0.5%, nuclei were pelleted by centrifugation (1 min, 12,000×***g***), and incubated for 30 min on ice in 10 mM HEPES, pH 7.5, containing 0.35 mM NaCl, 1 mM EDTA, 1 mM DTT and 0.2 mM PMSF. The samples were centrifuged (4°C, 5 min, 17,000×***g***) and protein concentrations in the supernatants were measured with Bradford assay.

The extracts of nuclear proteins (10 or 20 µg) were incubated for 30 min at room temperature in 15 µl of binding buffer (10 mM HEPES pH 7.5, 0.5% TritonX-100, 2.5% glycerol, 4 mM DTT) containing 1 µg of poly(dI-dC) and 40 fmoles of a fluorescently labeled double-stranded oligonucleotide probe for: NF-κB, STAT1/3, AP1 and Sp1 transcription factors. NF-κB and STAT1/3 probes were labeled with Cy5, AP1 probe – with Cy5 or 6FAM and Sp1 probe with 6FAM. The sequences of probes were as follows (binding sequences are underlined):

NF-κB: 5′-AGTTGAGGGGACTTTCCCAGGC-3′

AP-1: 5 ´-AGTGGCTTGATGACTCAGCCGGAA-3′

STAT1/3: 5′-AGTGACATTTCCCGTAAATCCA-3′

Sp1: 5′-AGTATTCGATCGGGGCGGGGCGAGC-3′.

To confirm specificity of binding, unlabeled competitive probes were included in the test. DNA-protein complexes were separated in a 5% non-denaturing polyacrylamide gel in Tris-boric acid-EDTA buffer, pH 8.0. Fluorescent signals were visualized using ChemiDoc MP Imaging System (Bio-Rad). The gels were then stained with Coomassie Brilliant Blue to confirm equal loading of protein. Densitometry was performed using Quantity One software (Bio-Rad). The fluorescent signals were normalized to the total amount of protein in each lane.

## Supplementary Material

Supplementary information
