# Supplementary material for: shRNAs targeting mouse Adam10 diminish cell response to proinflammatory stimuli independently of Adam10 silencing
Source: Biol Open. 2022 Mar 4;11(3):bio059092. doi: 10.1242/bio.059092 (PMC8905717; doi:10.1242/bio.059092)
Supplement: Supplementary information [file biolopen-11-059092-s1.pdf]

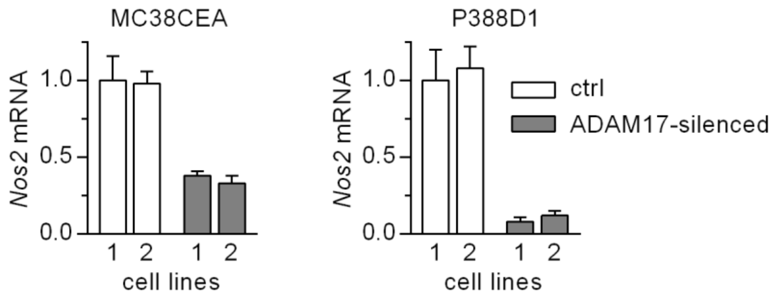

Fig. S1. RT-qPCR analysis of *Nos2* expression in MC38CEA and P388D1 cell lines, denoted as 1 and 2, derived from clones of cells, in which ADAM17 expression was silenced with a plasmid coding for shRNA targeting *Adam17* mRNA (sequence: 5'-AACGAATGCTGGTGTATAAGT-3', SuperArray Bioscience Corp., Maryland). Control cells (ctrl) were transfected with a plasmid coding for non-interfering shRNA (sequence 5'-GAAGATGCTATTAGAGCAATT-3', SuperArray Bioscience Corp.). *Nos2* expression was induced with IL1β + IFNγ (MC38CEA) or LPS (P388D1).

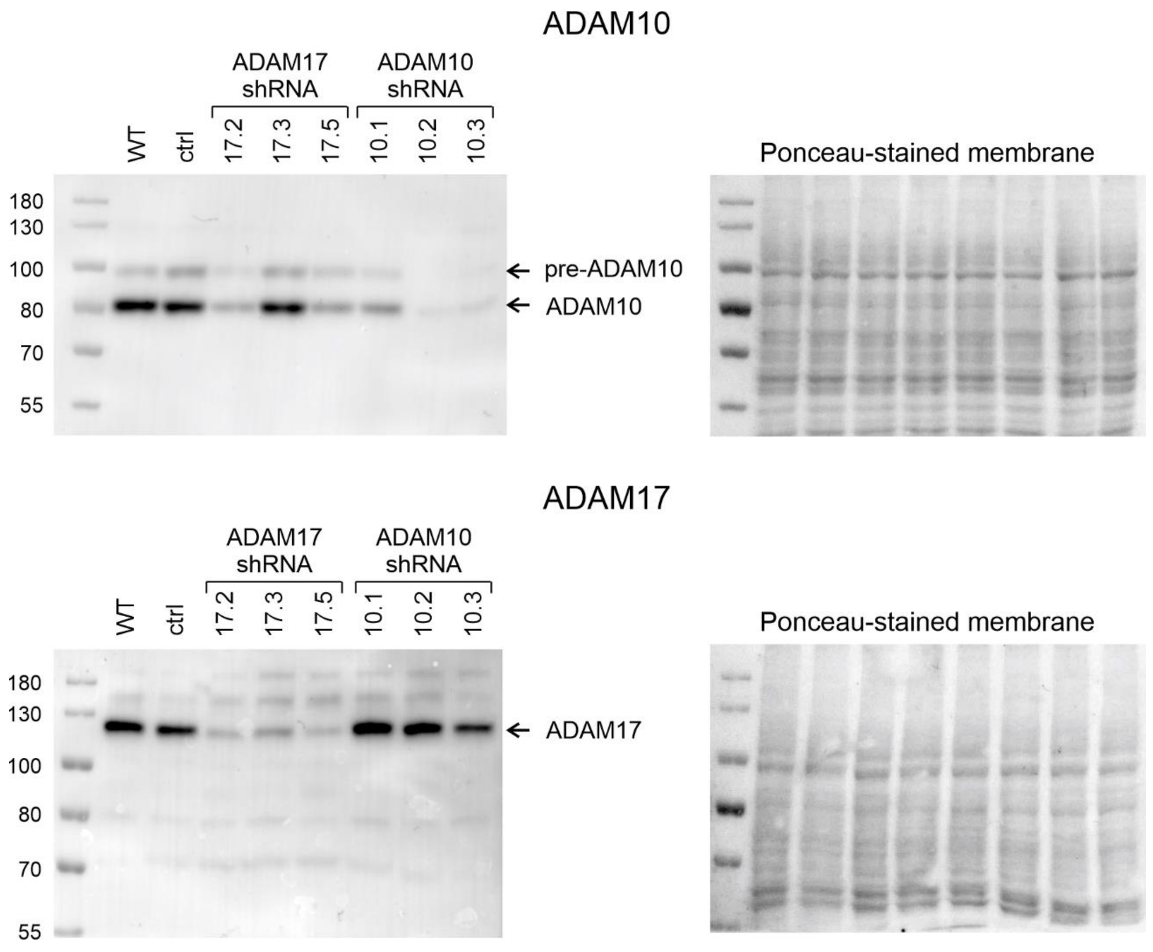

Fig. S2. Uncropped images of WB merged with protein ladder and Ponceau S stained membranes from Fig. 1B of the main text.

Biology Open • Supplementary information

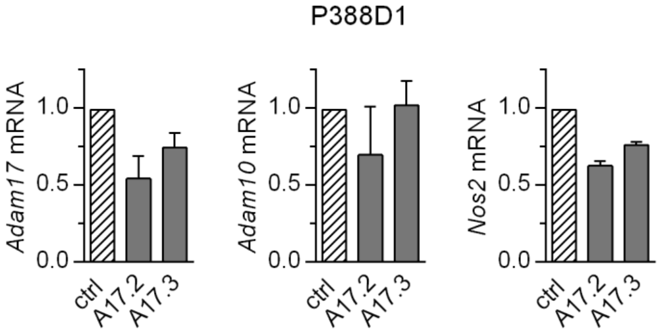

Fig. S3. RT-qPCR analysis of *Adam17*, *Adam10*, and *Nos2* expression in P388D1 cell lines generated by transduction of cells with leniviral vectors (MISSION®) encoding non-targeting shRNA (control, ctrl) or shRNAs targeting *Adam17* (sequence 2, A17.2 and sequence 3, A17.3). The relative levels of transcripts in control cells were taken as 1. Data presented as MV ± SD are from three independent experiments.

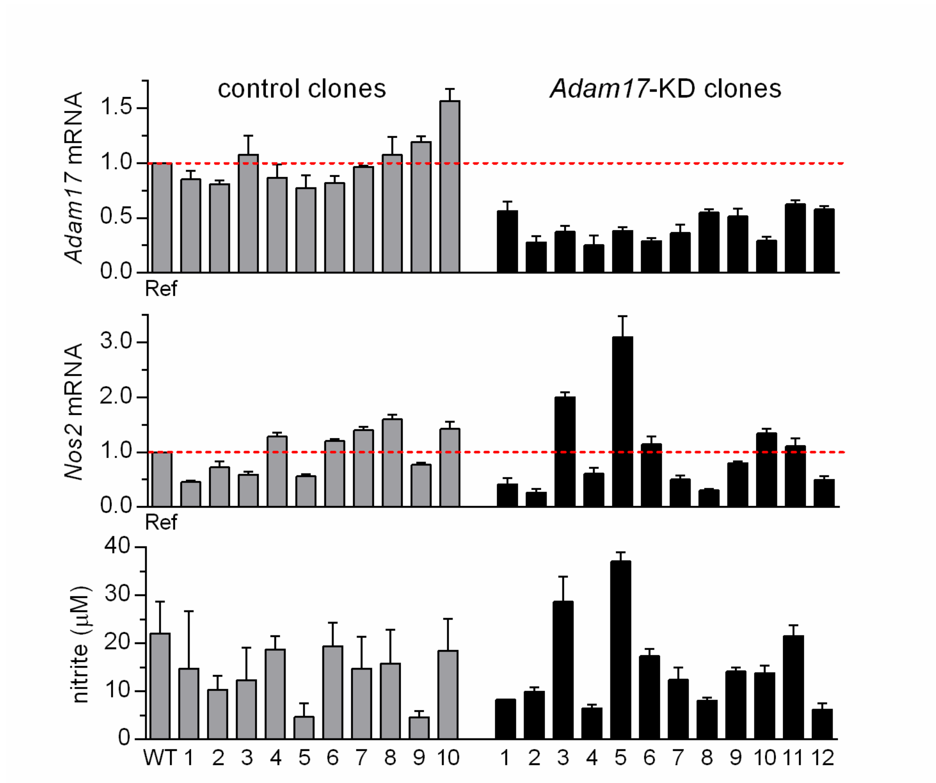

Fig. S4. Knockdown of *Adam17* expression using CRISPR/Cas9 gene editing did not affect iNOS expression and activity. RT-qPCR analysis of *Adam17* and *Nos2* (upper and middle panels) and levels of nitrite (lower panel) in cultures of individual MC38CEA cell clones derived from the control or *Adam17*-knockdown populations described in Fig. 1d of the main text. The cells were stimulated with IL1β and IFNγ for 6 h (RT-qPCR) or 18 h (nitrite). The average of relative levels of *Adam17* and *Nos2* transcripts in all control clones (Ref) were taken as 1. MV ± SD from two independent experiments are shown.

Biology Open • Supplementary information

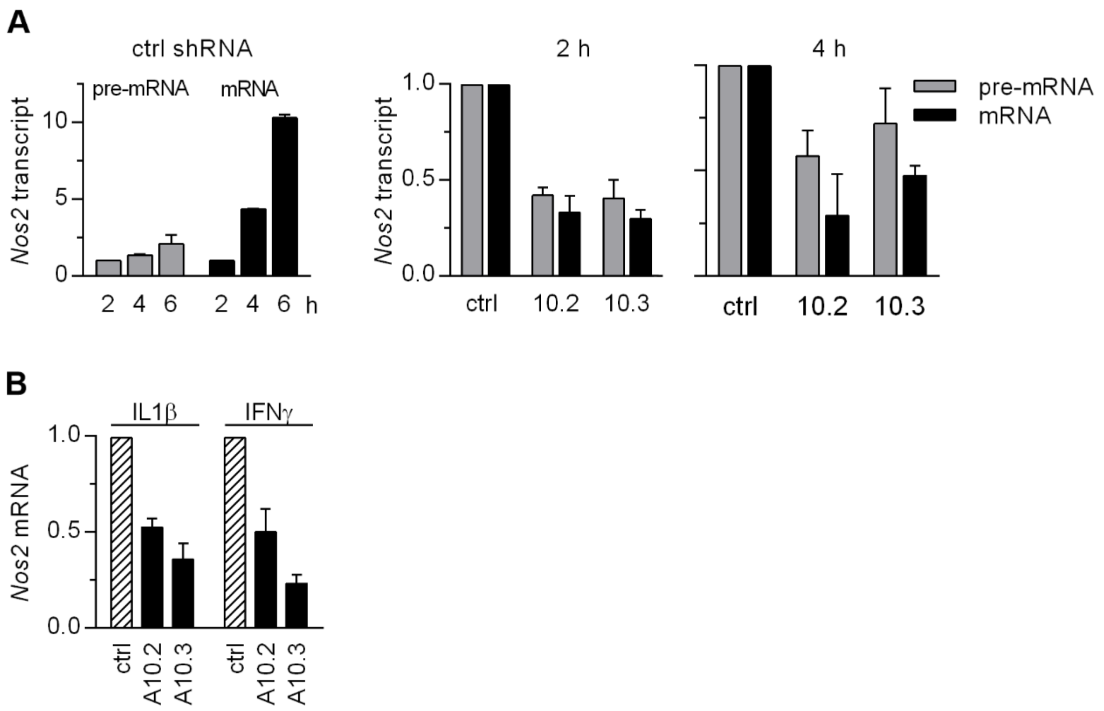

Fig. S5. The influence of shRNAs on *Nos2* levels is fast and independent of the type of cytokine stimulus. RT-qPCR analysis of *Nos2* transcript levels in MC38CEA cells expressing non-targeting shRNA (ctrl), or ADAM10 targeting shRNAs: A10.2 or A10.3. (A) The cells were treated with IL1β or IFNγ for indicated times before RNA isolation. The left panel show kinetics of increase of *Nos2* transcript levels in control cells. The relative levels of *Nos2* transcript in control cells after 2 h-stimulation with cytokines were taken as 1. For right panels the relative levels of *Nos2* transcripts in control cells were taken as 1. (B) The cells were treated with IL1β or IFNγ for 6 h prior to RNA isolation. The relative levels of *Nos2* mRNA in control cells were taken as 1. Data are presented as MV ± SD from two (A) or three (B) independent experiments.

Biology Open • Supplementary information

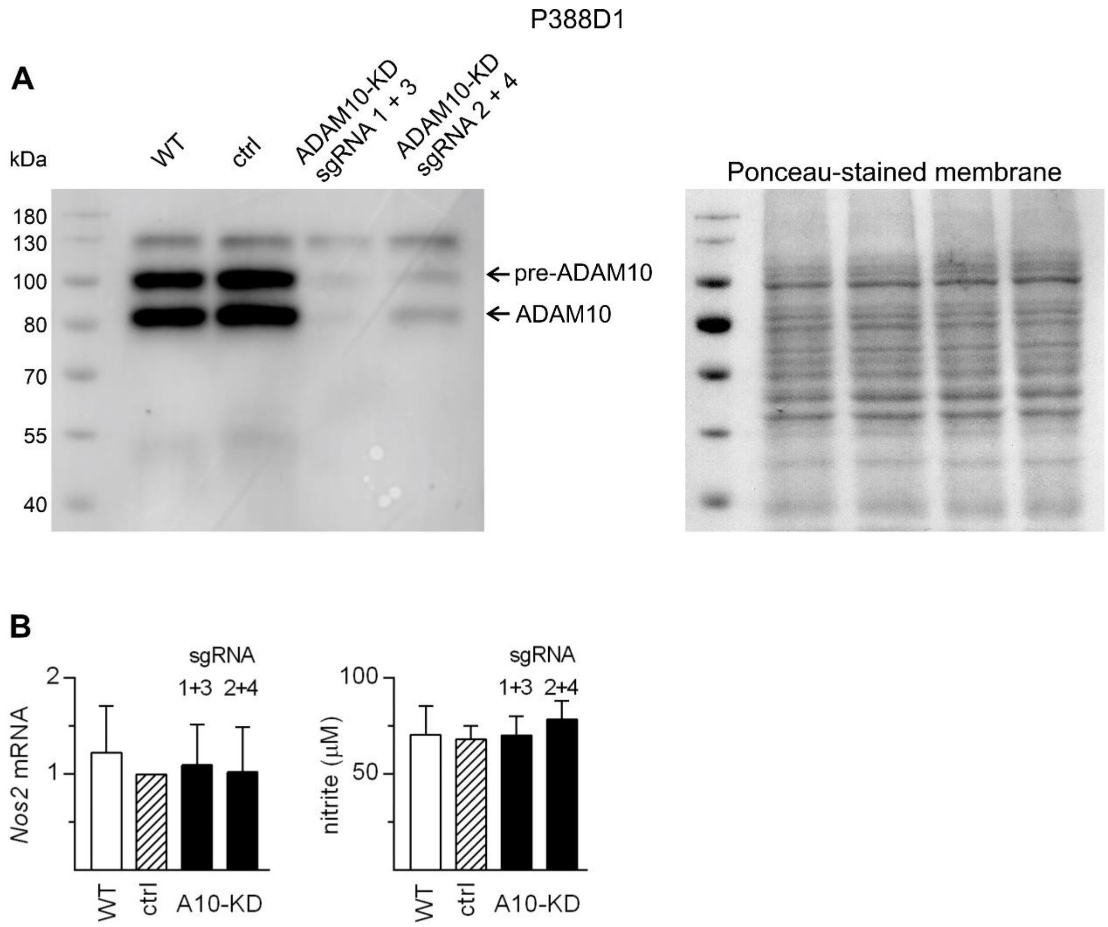

Fig. S6. Knockdown of *Adam10* expression using CRISPR/Cas9 gene editing did not affect iNOS expression and activity in P388D1 cells. (A) WB analysis of ADAM10 in P388D1 cells, in which *Adam10* expression was knocked down by CRISPR/Cas9 using pairs of sgRNAs 1+3 or 2+4 (sequences are given in Supplementary Table 3). In control cells *EGFP*-specific sgRNA was used instead of *Adam10*-specific sgRNAs. Representative WB image (merged with protein ladder) and Ponceau S stained membrane of 2 independent experiments are presented. (B) RT-qPCR analysis of *Nos2* mRNA levels and measurement of nitrite levels in lysates and media, respectively, of P388D1 cells described in (A), stimulated with LPS for 6 h (RT-qPCR) or 20 h (nitrite). Data are presented as MV ± SD from three (RT-qPCR) or four (nitrite concentration measurement) independent experiments.

Biology Open • Supplementary information

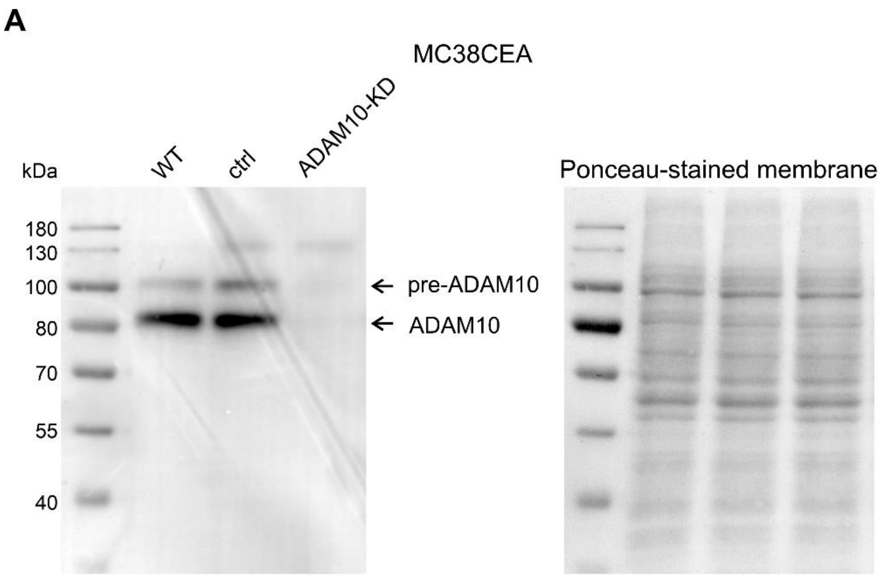

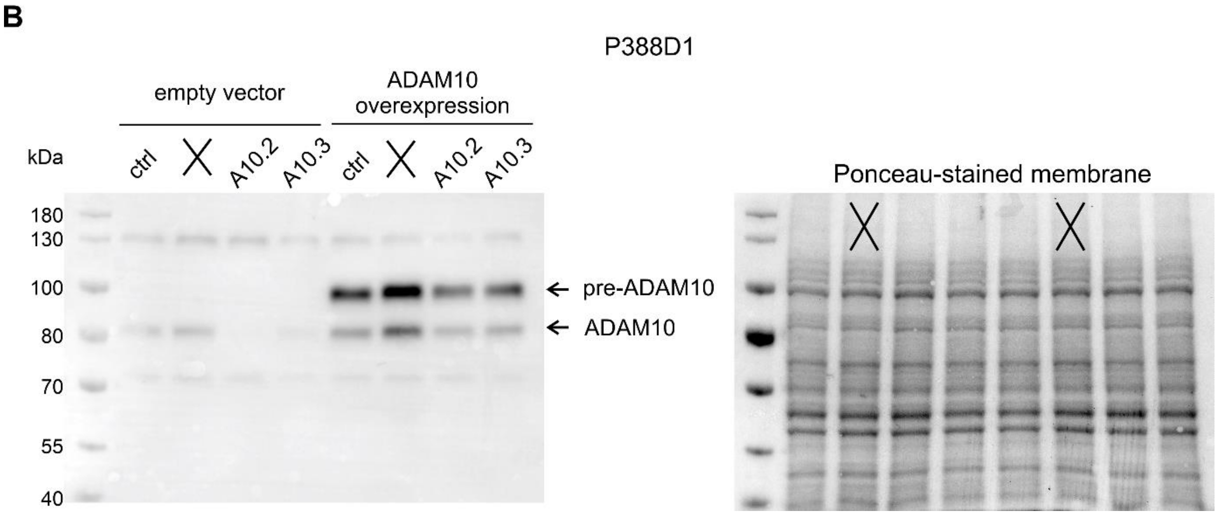

Fig. S7. Uncropped images of WB merged with protein ladder and Ponceau S stained membranes from Fig. 2A (A) and 2D (B) of the main text. In panel (B) crosses indicate lanes that were removed from the image in the Fig. 2D.

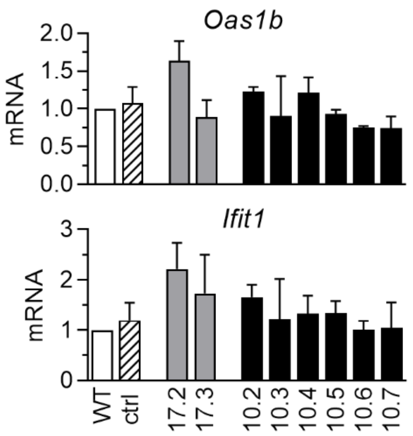

Fig. S8. Analyzed shRNAs did not induce interferon response. The levels of transcripts encoding interferon-inducible 2'-5'-oligoadenylate synthase 1b (*Oas1b*) and interferon induced protein with tetratricopeptide repeats 1 (*Ifit1*) were not markedly increased in MC38CEA cells expressing shRNAs as analyzed by qRT-PCR. MV ± SD from two independent experiments are shown.

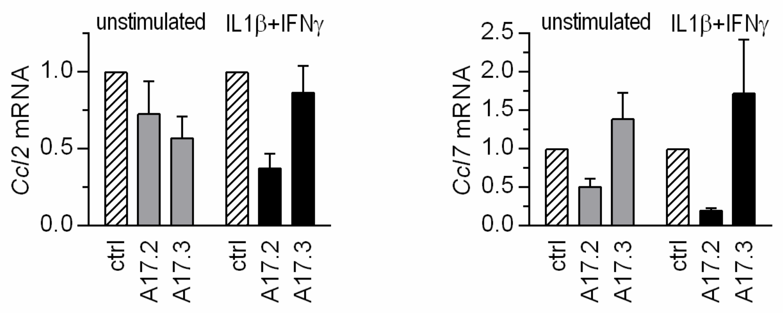

Fig. S9. RT-qPCR analysis of *Ccl2* and *Ccl7* mRNA levels in MC38CEA cells expressing not-targeting shRNA (ctrl), or A17.2, or A17.3 shRNAs. The cells were left untreated or were stimulated with IL1β and IFNγ for 6 h prior to RNA isolation. The relative levels of the transcripts in control cells were taken as 1. MV ± SD from two independent experiments are shown.

Biology Open • Supplementary information

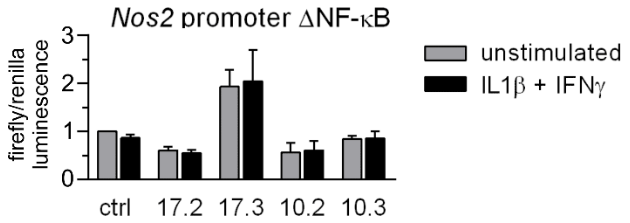

Fig. S10. Relative luminescence signals in MC38CEA cells transduced with one of lentiviral vectors encoding non-targeting shRNA (ctrl) or one of the *Adam17- or Adam10*-targeting shRNA sequences and then transfected with a plasmid containing luciferase CDS under *Nos2* promoter deprived of NF-κB binding sites (ΔNF-κB). The cells were left unstimulated or were stimulated with IL1β and IFNγ for 6 h. Luminescence signals of unstimulated, control cells were taken as 1. Data are shown as MV ± SD from four independent experiments.

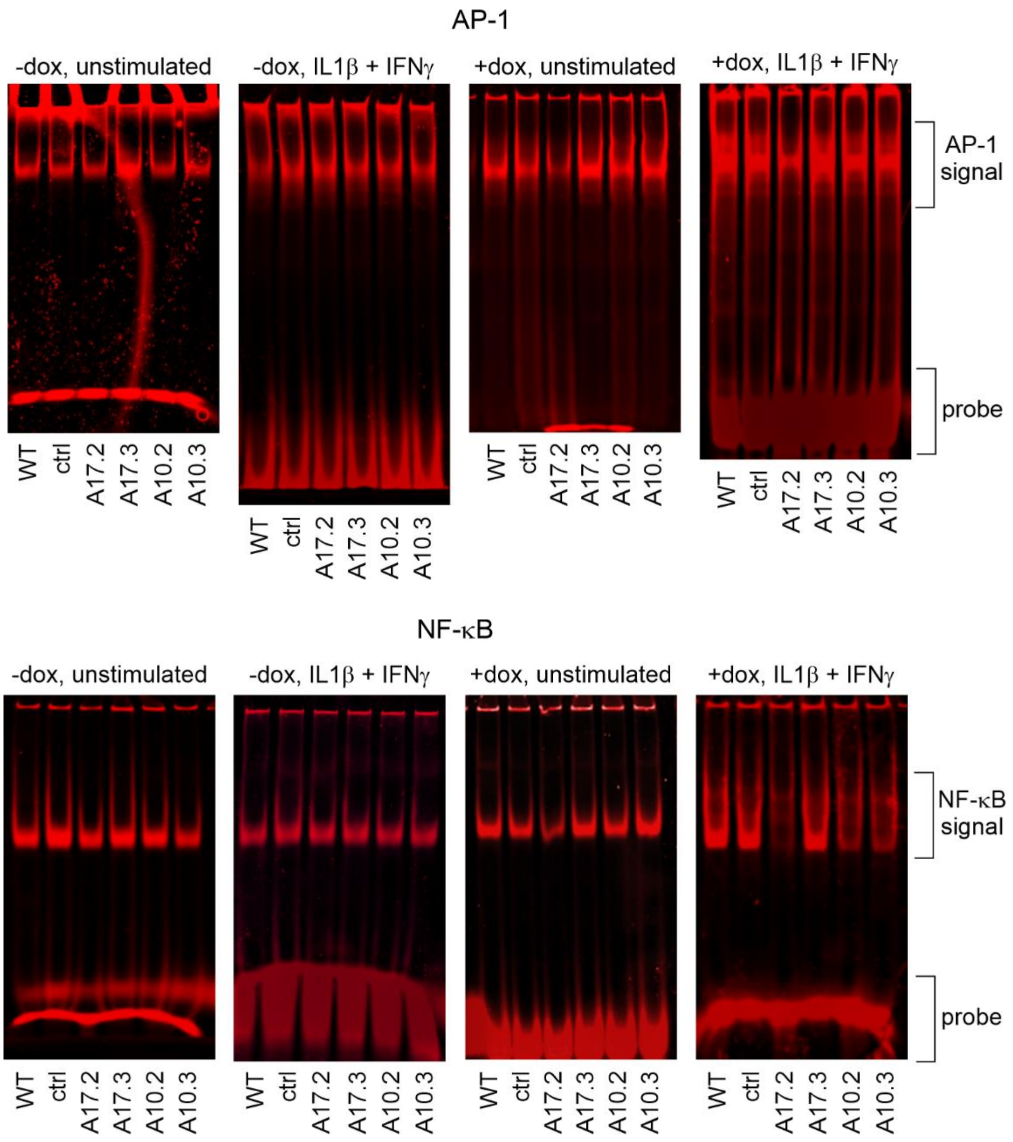

Fig. S11. Uncropped images of EMSA results presented in Fig. 4C. The time of electrophoreses was not exactly the same and hence the differences in the distance traveled by the probes and the transcription factor-probe complexes.

Biology Open • Supplementary information

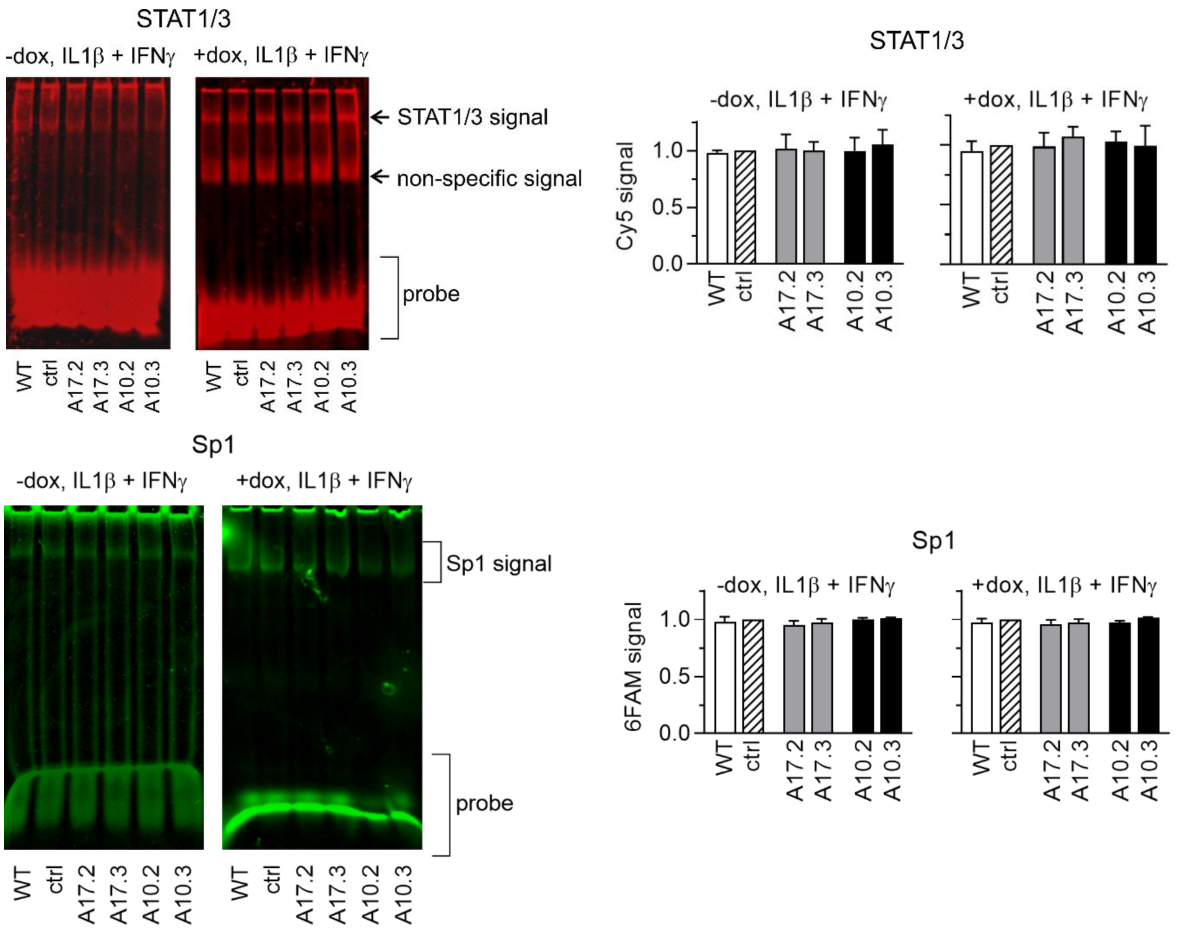

Fig. S12. Expression of studied shRNAs did not affect STAT1 and Sp1 interactions with specific DNA sequences in nuclear extracts of cytokine-stimulated MC38CEA cells. Left panels show representative uncropped images of EMSA results. Right panels present relative fluorescence signals of STAT1/3- or Sp1-bound probes in nuclear extracts of the cells, in which expression of shRNAs was induced by doxycycline. The cells were stimulated with cytokines for 30 min prior to nuclear protein isolation. Data are from three (STAT) or two (Sp1) independent experiments.

Table S1. Sequences targeted by shRNAs used in this study

	**Targeted sequence**	**Targeted region**	**Catalog no***
ctrl**	CAACAAGATGAAGAGCACCAA	NA	SHC002
17.2	GCGACACACTTAGAAACATTA	*Adam17* CDS	TRCN0000031953
17.3	CGCGACTTGAGAAGCTTGATT	*Adam17* CDS	TRCN0000031949
17.5	CCCTTGAAGAATACTTGTAAA	*Adam17* CDS	TRCN0000031951
10.1	CAGCTCTATATCCAGACAGAT	*Adam10* CDS	TRCN0000031847
10.2	GCAGAGAGATACATTAAAGAT	*Adam10* CDS	TRCN0000031844
10.3	CCAGGAGAGTCTAAGAACTTA	*Adam10* CDS	TRCN0000031848
10.4	CCATGTTTGCTGCATGAAGAA	*Adam10* CDS	TRCN0000031845
10.5	GAGTTATCAAATGGGACACAT	*Adam10* CDS	TRCN0000031846
10.6	GGCACAAAGTCTTAGAATATT	*Adam10* 3'UTR	NA
10.7	CCAGCTACATCACTTAAATTA	*Adam10* 3'UTR	NA

*The RNAi Consortium library (available via Merck); **non-targeting control;
NA – not applicable

Table S2. Summary of activities of studied shRNAs

	Name	Specific towards	ADAM17 levels	ADAM10 levels	Expression** of		
					Nos2	*Ccl2*	*Ccl7*
expressed constitutively	A17.2	ADAM17	↓	↓	↓	↓	↓
	A17.3	ADAM17	↓	↑	↑	↑	↑
	A17.5	ADAM17	↓	↓	↓	ND	ND
	A10.1	ADAM10	–*	↓	↓	ND	ND
	A10.2	ADAM10	–	↓	↓	↓	↓
	A10.3	ADAM10	–	↓	↓	↓	↓
	A10.4	ADAM10	–	↓	–	–/↑	–
	A10.5	ADAM10	–	↓	↓	↓	↓
	A10.6	ADAM10	–	↓	↓	↓	↓
	A10.7	ADAM10	–	↓	↓	↓	↓
dox-inducible	A17.2	ADAM17	↓	↓	↓	↓	ND
	A17.3	ADAM17	↓	–/↑	↑	–/↑	ND
	A10.2	ADAM10	–	↓	↓	↓	ND
	A10.3	ADAM10	–	↓	–	↑	ND

*„–” – not changed; ND – not determined; **expression in the cells stimulated with IL1β+IFNγ

Table S3. List of primers used for RT-qPCR or PCR

Gene	Primer forward	Primer reverse
Eef2	CCACGGCAAGTCCACGCTGAC	AGAAGAGGGAGATGGCGGTGGATT
Polr2b	GGATTCTGGGAACGTCGGAG	CCGGAGTGATCTCATCGTCG
Nos2	AAGGCCAAACACAGCATACC	CTGAAGCACTAGCCAGGGAC
Nos2 pre-mRNA	GCTCCTCAAGCCTGGTCTTT	GGGCATTTAGGCAGGAGTGT
Adam17	AGGGTTCTAGCCCACATAGGA	TGGAGACTGCAAACGTGAAA
Adam10	CCGGGCTCTCCATGTAATGA	CCAGTGAGCCACAATCCAC
Ccl2	AGCACCAGCCAACTCTCACT	GCTGCTGGTGATCCTCTTGT
Ccl7	CTTCTGTGCCTGCTGCTCATA	TCCATGCCCTTCTTTGTCTTGA
Oas1b	AGGGCCTCTAAAGGGGTCAA	ACCTCGCACAGCTGTTTCTT
Ifit1	GCTCTGCTGAAAACCCAGAGA	AAGGAACTGGACCTGCTCTGA
Mycoplasma sp. 16S rDNA	ACTCCTACGGGAGGCAGCAGTA	TGCACCATCTGTCACTCTGTTAACCTC

Table S4. Oligonucleotides encoding sgRNAs targeting *Adam10*

Name	Targeted region and sequence*	Oligonucleotides
10-1	exon 2: CAAACGAGCAGTCTCACATG**AGG**	Top: CACCGCAAACGAGCAGTCTCACATG<br>Bottom: AAACCATGTGAGACTGCTCGTTTGC
10-2	exon 3: ATGTCCAGTGTAAATATGAG**AGG**	Top: CACCGATGTCCAGTGTAAATATGAG<br>Bottom: AAACCTCATATTTACACTGGACATC
10-3	exon 4: GTTTCATCAAGACTCGTGG**TGG**	Top: CACCGGTTTCATCAAGACTCGTGG<br>Bottom: AAACCCACGAGTCTTGATGAAACC
10-4	exon 5: CCCATAAATACGGCCCACAG**GGG**	Top: CACCGCCCATAAATACGGCCCACAG<br>Bottom: AAACCTGTGGGCCGTATTTATGGGC

*PAM sequence is in bold
